# Characterization of Distinct T Cell Receptor Repertoires in Tumor and Distant Non-tumor Tissues from Lung Cancer Patients

**DOI:** 10.1016/j.gpb.2018.10.005

**Published:** 2019-08-31

**Authors:** Xiang Wang, Botao Zhang, Yikun Yang, Jiawei Zhu, Shujun Cheng, Yousheng Mao, Lin Feng, Ting Xiao

**Affiliations:** 1 State Key Laboratory of Molecular Oncology, Department of Etiology and Carcinogenesis, National Cancer Center/National Clinical Research Center for Cancer/Cancer Hospital, Chinese Academy of Medical Sciences and Peking Union Medical College, Beijing 100021, China; 2 Department of Thoracic Surgery, National Cancer Center/National Clinical Research Center for Cancer/Cancer Hospital, Chinese Academy of Medical Sciences and Peking Union Medical College, Beijing 100021, China

**Keywords:** Adaptive immune response, T cell receptor repertoire, Lung cancer, High-throughput sequencing, TCR diversity

## Abstract

T cells and T cell receptors (TCRs) play pivotal roles in **adaptive immune responses** against tumors. The development of next-generation sequencing technologies has enabled the analysis of the TCRβ repertoire usage. Given the scarce investigations on the TCR repertoire in **lung cancer** tissues, in this study, we analyzed TCRβ repertoires in lung cancer tissues and the matched distant non-tumor lung tissues (normal lung tissues) from 15 lung cancer patients. Based on our results, the general distribution of T cell clones was similar between cancer tissues and normal lung tissues; however, the proportion of highly expanded clones was significantly higher in normal lung tissues than in cancer tissues (0.021% ± 0.002% *vs.* 0.016% ± 0.001%, *P* = 0.0054, Wilcoxon signed rank test). In addition, a significantly higher **TCR diversity** was observed in cancer tissues than in normal lung tissues (431.37 ± 305.96 *vs.* 166.20 ± 101.58, *P* = 0.0075, Mann-Whitney U test). Moreover, younger patients had a significantly higher TCR diversity than older patients (640.7 ± 295.3 *vs.* 291.8 ± 233.6, *P* = 0.036, Mann-Whitney U test), and the higher TCR diversity in tumors was significantly associated with worse cancer outcomes. Thus, we provided a comprehensive comparison of the TCR repertoires between cancer tissues and matched normal lung tissues and demonstrated the presence of distinct T cell immune microenvironments in lung cancer patients.

## Introduction

Adaptive immune responses against tumors are promising prognostic indicators for multiple cancers [1]. T cells infiltrating the tumor microenvironment and their corresponding receptors play vital roles in adaptive immune responses. T cell responses to cancer cells depend largely on the affinity between T cell receptors (TCRs) and peptide-major histocompatibility complex (pMHC). Developing and maintaining highly diversified TCR repertoires to defend against numerous foreign pathogens is demanding [2]. TCRs are heterodimers composed of either specific α and β chains, representing the most common types of TCRs, or specific γ and δ chains. TCR diversity is characterized by recombination of the V/J gene segments of the TCRα and V/D/J gene segments of the TCRβ. The recombination particularly takes place in complementarity determining region 3 (CDR3) domain of TCR [3], [4]. Therefore, characterizing the connection between cancer cells and the host adaptive immune system, especially pertaining to the TCR CDR3 domain, is vital for understanding tumor immunology, particularly for identifying therapeutic targets and monitoring immunotherapy responses [5].

The development of next-generation sequencing (NGS) technologies has enabled detailed profiling of the immune system. Recently, advancements in platforms have facilitated the analysis of the TCR repertoire [6], especially TCRβ CDR3 sequencing, making it possible to track dominant TCR clones in different tissues over time [7], [8], [9]. Tumor heterogeneity at the genetic level is often connected with strong diversity in tumor infiltrating lymphocytes (TILs) within tumor lesions [10], [11]. The clonal TIL composition can be assessed by analyzing their TCR repertoires [12]. Accordingly, studies on the spatial heterogeneity of TILs have been reported to elucidate the changes in intratumoral and peripheral T cells in several cancers, including renal cell carcinoma [13], esophageal squamous cell carcinoma [14], primary liver carcinoma [15], and lung adenocarcinoma [16].

A recent study [17] analyzed TCR and B cell receptor (BCR) repertoires in sorted cell subsets of tumor, distant non-tumor tissue (NT), and peripheral compartments (blood/draining lymph node) from 47 non-small cell lung cancer (NSCLC) patients and identified distinct adaptive immune responses in NSCLC. The presence of tertiary lymphoid structures (TLSs) in the microenvironment of lung cancer also enhanced the T cell clonal expansion in tumors. However, the relationship between the diversity of TCR clones and the clinical features of the lung cancer patients has not been further explored. Here, we compared the frequency of T cell clones and the clonal diversity of TCR repertoires in lung cancer tissues and the matched normal lung tissues to elucidate the association between TCR diversity and the prognosis of lung cancer patients.

## Results

### Global profile of the TCR repertoire sequencing data

**Figure 1:**
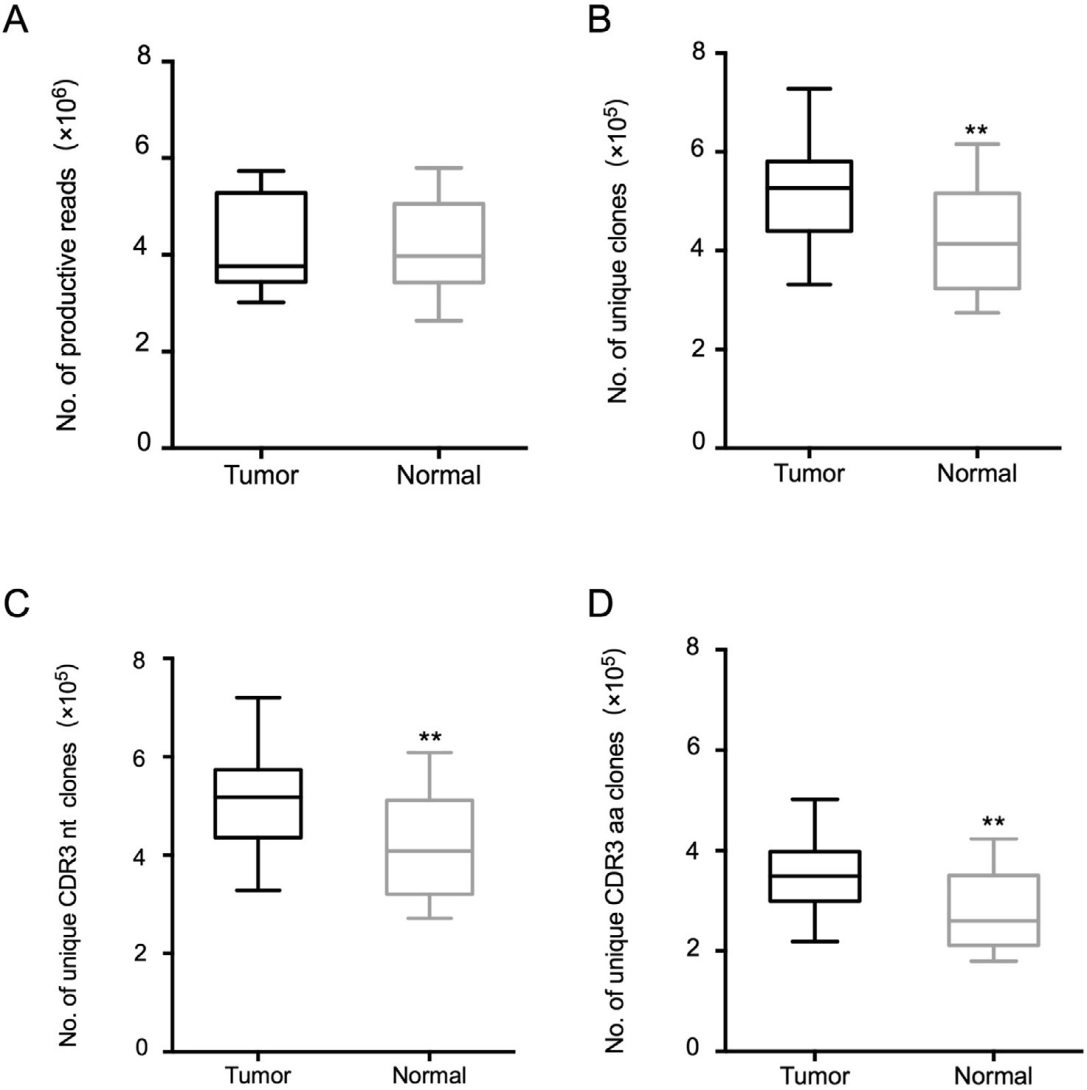
**Statistical characteristics of the TCR repertoires sequencing data** **A.** Distribution of the productive reads in 15 tumor tissues and 15 non-tumor (normal) tissues. **B.** Distribution of unique reads in tumor tissues and non-tumor tissues. **C.** Numbers of unique CDR3 nt clones in tumor tissues and non-tumor tissues. **D.** Numbers of unique CDR3 aa clones in tumor tissues and non-tumor tissues. (Wilcoxon signed rank test, **, *P* < 0.01; ns, not significant). TCR, T cell receptor; CDR, complementarity determining region; nt, nucleotide; aa, amino acid.

To assess the TCR repertoire in the tumor tissues and normal tissues of patients with lung cancer, we obtained RNA from 30 paired specimens isolated from the 15 patients and performed TCRβ sequencing by amplifying the TCRβ CDR3 region, a method that we had previously used [15], [18]. Detailed information about the TCRβ repertoire data is included in Table S1. We obtained a total of 125,075,908 productive TCRβ reads (sequence of the read is in frame and does not have a premature stop codon), with an average of 4,169,197 reads per sample. In tumor tissues, 3,015,213–5,733,528 productive reads were obtained, and 331,272–727,815 unique clones were identified, whereas 2,639,987–5,797,795 productive reads and 274,202–615,647 unique clones were obtained in the normal lung tissues. The distribution of the productive reads in tumor tissues and normal lung tissues was similar (*P* = 0.978, Figure 1A), but the distribution of unique clones differed significantly between the two tissues (*P* = 0.0054, Wilcoxon test). The number of unique clone reads in tumor tissues was much higher (523,928 ± 112,511, *P* = 0.0054, Wilcoxon test) than that in normal tissues (417,735 ± 102,716) (Figure 1B), indicating that the tumor tissues contained more T cell clonotypes than the normal lung tissues. We also compared the numbers of unique clones based on the nucleotide (nt) sequences and amino acid (aa) sequences of CDR3 between tumor tissues and normal lung tissues (Figure 1C and D). We found that both the unique CDR3 nt clones (517,687 ± 110,391, *P* = 0.0054, Wilcoxon test) and unique CDR3 aa clones (352,028 ± 79,534, *P* = 0.0043, Wilcoxon test) were significantly more abundant in the tumor tissues than those in the non-tumor tissues (CDR3 nt: 412,976 ± 101658, CDR3 aa: 275439 ± 71131).

### A higher frequency of highly expanded clones in normal lung tissues than in tumor tissues

**Supplementary Figure S1:**
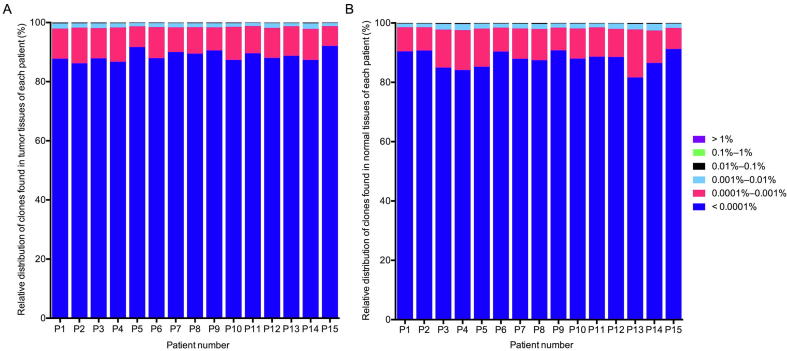
**The general frequency of T cell clones in tumor tissues and normal tissues A.** Distribution of total T cell clones in tumor tissues. **B.** Distribution of total T cell clones in normal tissues.

The overall composition of TCRβ CDR3 clones with different frequencies is summarized in Table S2. The general frequencies of T cell clones were quite similar between cancer tissues and normal lung tissues, as shown in Figure S1. The percentage of clones with frequency <0.0001% ranged from 86.20% to 92.11% (88.77 ± 0.46%) in tumor tissues and from 81.64% to 91.22% (87.77 ± 0.73%) in normal lung tissues. We also calculated the frequency of highly expanded clones (HECs) (frequency >0.1%), which ranged from 0.007% to 0.027% (0.016 ± 0.001%) in tumor tissues and from 0.011% to 0.038% (0.021 ± 0.002%) in normal lung tissues (*P* = 0.0054, Wilcoxon test). These data indicate that most T cell clones in cancer tissues and normal lung tissues had extremely low frequencies, and only a small proportion of T cell clones were HECs.

**Figure 2:**
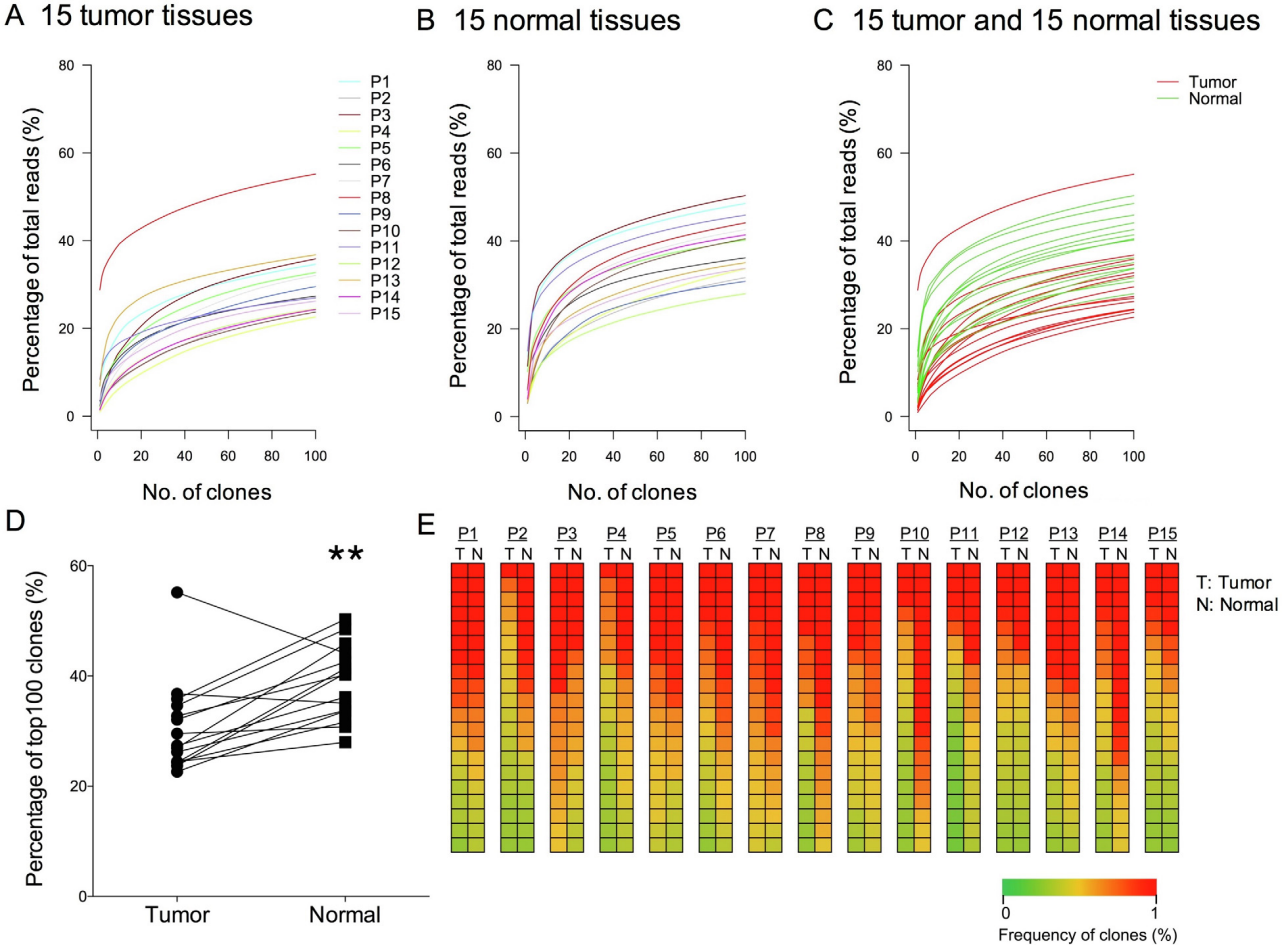
**Distribution of T cell clones in the TCR repertoires in tumor tissues and normal tissues** **A.** Cumulative frequencies of the TOP100 T cell clones in the 15 tumor tissues. **B.** Cumulative frequencies of the TOP100 T cell clones in the 15 normal tissues. **C.** Cumulative frequencies of the TOP100 T cell clones in the 15 tumor and the matched normal tissues (tumor and normal tissues are indicated in red and green, respectively). **D.** Percentage of the TOP100 T cell clones in tumor and normal tissues. **E.** Heatmaps of the 20 T cell clones with the highest frequency in tumor tissues or normal tissues from each of the 15 patients. **, *P* < 0.01; Wilcoxon signed rank test. TCR, T cell receptor; T, tumor tissue; N, normal tissue.

To compare HECs between cancer tissues and normal lung tissues, we examined the commonality of the 100 most abundant T cell clones (TOP100 clones), which accounted for approximately 0.1% of all T cell clones in each sample. To this end, we first calculated and plotted the cumulative frequencies of the TOP100 clones (Figure 2). Based on the cumulative frequency curves, the cumulative distribution of the TOP100 clones in both tissues displayed the same trend (Figure 2A and B). However, when the TOP100 clones in the two tissues were merged, most of the cumulative frequencies in normal lung tissues seemed higher than those in tumor tissues (Figure 2C). The cumulative frequency of the TOP100 clones was significantly higher in normal lung tissues (38.82 ± 6.74, *P* = 0.0043, Wilcoxon test) than in tumor tissues (30.45 ± 8.29) (Figure 2D). Heatmaps of the 20 clones with the highest frequency in each pair of samples (Figure 2E) also demonstrated the presence of more HECs in normal lung tissues than in tumor tissues.

### A higher T cell clonal diversity in tumor tissues than in normal lung tissues

**Figure 3:**
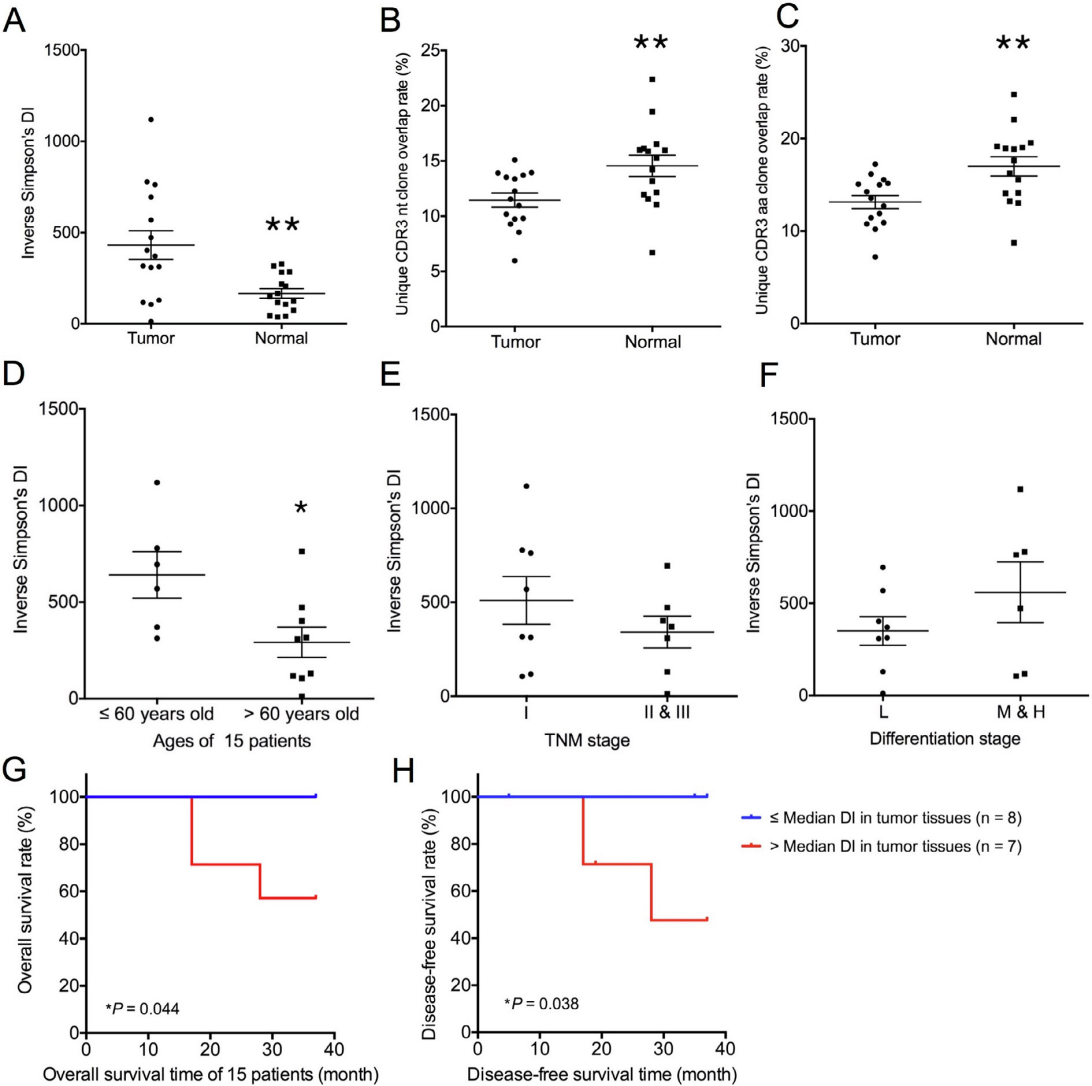
**T cell clonal diversity in tumor tissues and normal tissues** **A.** Distribution of T cell clonal diversity in tumor tissues and normal tissues based on inverse Simpson’s DIs. **B.** CDR3 nt sequence overlap rates in tumor tissues and normal tissues. **C.** CDR3 aa sequence overlap rates in tumor tissues and normal tissues. **D.** Inverse Simpson’s DI accordingly in terms of patient age. **E.** Inverse Simpson’s DI accordingly in terms of TNM stage. **F.** Inverse Simpson’s DI accordingly in terms of tumor differentiation stages. **G.** Kaplan-Meier plots for the overall survival time of the 15 patients based on TCR diversity. **H.** Kaplan-Meier plots for disease-free survival time of the 15 patients based on TCR diversity. *, *P* < 0.05; **, *P* < 0.01; Mann-Whitney U test. DI, diversity index; CDR3, complementarity determining region 3; nt, nucleotide; aa, amino acid.

**Supplementary Figure S2:**
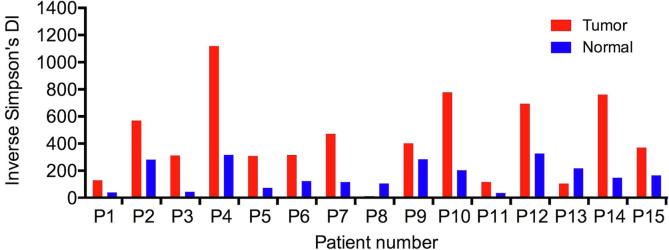
**The inverse Simpson’s DI in tumor tissues and normal tissues for each patient**

We analyzed the differences between tumor tissues and normal lung tissues from 15 patients based on the diversity of T cell clones. To this end, we calculated the inverse Simpson’s diversity index (DI) values to determine the diversity of T cell clones: high values indicate even distribution of TCR clones, and low values indicate enrichment of T cell clones. On average, the inverse Simpson’s DI was 431.37 (range: 11.75–1118.66) in tumor tissues and 166.20 (range: 36.96–327.83) in normal lung tissues (Figure 3A). A significantly higher T cell clonal diversity was observed in tumor tissues (431.37 ± 305.96, *P* = 0.0075, Mann-Whitney U test) than in normal lung tissues (166.20 ± 101.58) based on all results. This trend was observed in all patients except patient 8 and patient 13 (Figure S2).

**Supplementary Figure S3:**
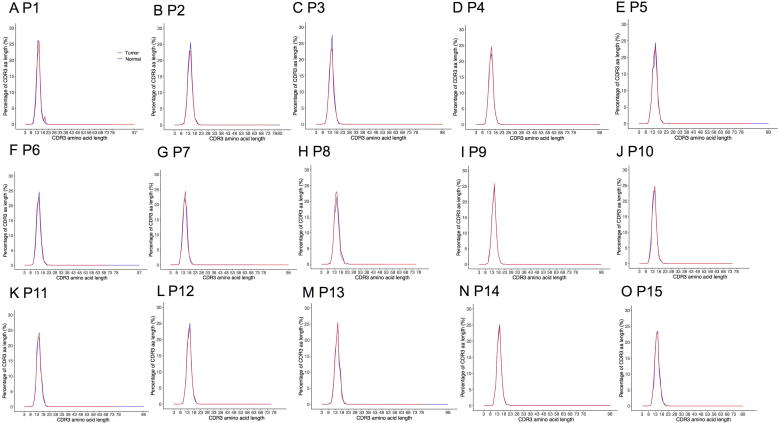
**The TCR CDR3 length distribution in tumor tissues and distant lung tissues from 15 patients** The X axis indicates the length of CDR3 (aa); the Y axis represents the percentage of CDR3 fragments at the indicated length among all the CDR3 fragments.

Furthermore, to compare the TCR repertoire similarity for each patient, we calculated sequence overlap rates in the two tissue types (overlap rates represent the percentage of similar clones between different patients or different tissues). In our study, shared clones refer to CDR3 nt or CDR3 aa clones that were same in the two tissues. The average CDR3 nt sequence overlap rate for the 15 patients was 11.46% (range: 5.96–15.09%) in tumor tissues and 14.57% (range: 6.70–22.39%) in normal lung tissues (Figure 3B), whereas the average CDR3 aa sequence overlap rate for the 15 patients was 13.14% (range: 7.19–17.23%) in tumor tissues and 16.99% (range: 8.73–24.76%) in normal lung tissues (Figure 3C). Therefore, the overlap rates were significantly higher in normal lung tissues (16.99% ± 4.02%, *P* = 0.0057, Mann-Whitney U test). Besides, the CDR3 aa lengths in most samples range from 8–23 aa, with a peak length of 15 aa. The CDR3 length distributions in both tissue types were quite similar (Figure S3).

### Association of TCR diversity with clinicopathological characteristics

**Supplementary Figure S4:**
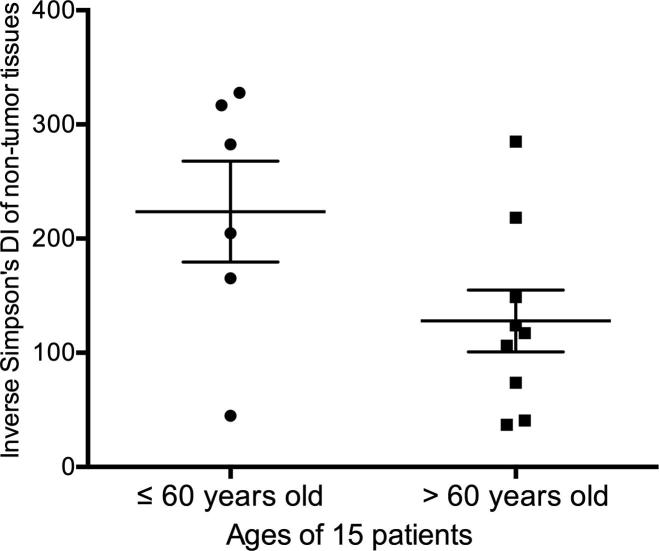
**The T cell clonal diversity in normal tissues in patients grouped by age**

**Supplementary Figure S5:**
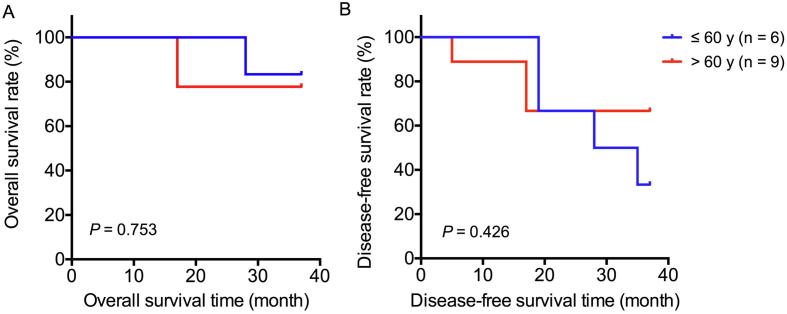
**Kaplan-Meier plots for survival time of the 15 patients based on age A.** Kaplan-Meier plots for overall survival time of the 15 patients based on age. **B.** Kaplan-Meier plots for disease-free survival time of the 15 patients based on age.

Potential correlations were examined between the TCR diversity in tumor tissues and normal lung tissues and patient age (≤60 years old or >60 years old), tumor TNM stage, and tumor differentiation. Younger patients (age ≤60 y) had higher inverse Simpson’s DIs in tumor tissues (640.7 ± 295.3, *P* = 0.0360, Mann-Whitney U test, Figure 3D). In normal tissues, we observed a trend that younger patients had higher inverse Simpson’s DIs than older patients, but the difference was not significant (*P* = 0.0879, Mann-Whitney U test, Figure S4). Furthermore, no significant differences were observed regarding the associations of the TCR diversity with tumor TNM stage and differentiation stage (Figure 3E and F). Using the median value of inverse Simpson’s DIs in tumor tissues as the cutoff, we classified the 15 patients into two groups and found a significant difference in prognosis. The higher inverse Simpson’s DI values in tumor tissues were associated with worse cancer outcomes. As shown in Figure 3G and H, significant longer survival time was observed in patients with lower DI (*P* = 0.044 for overall survival time and *P* = 0.038 for disease-free survival time). Since TCR diversity was associated with age, we also compared the survival time of the two groups of patient aged above or below 60 years old. However, we found no correlations between age and cancer outcome (Figure S5).

### Similar usage of V and J gene segments in tumor tissues and normal lung tissues

**Figure 4:**
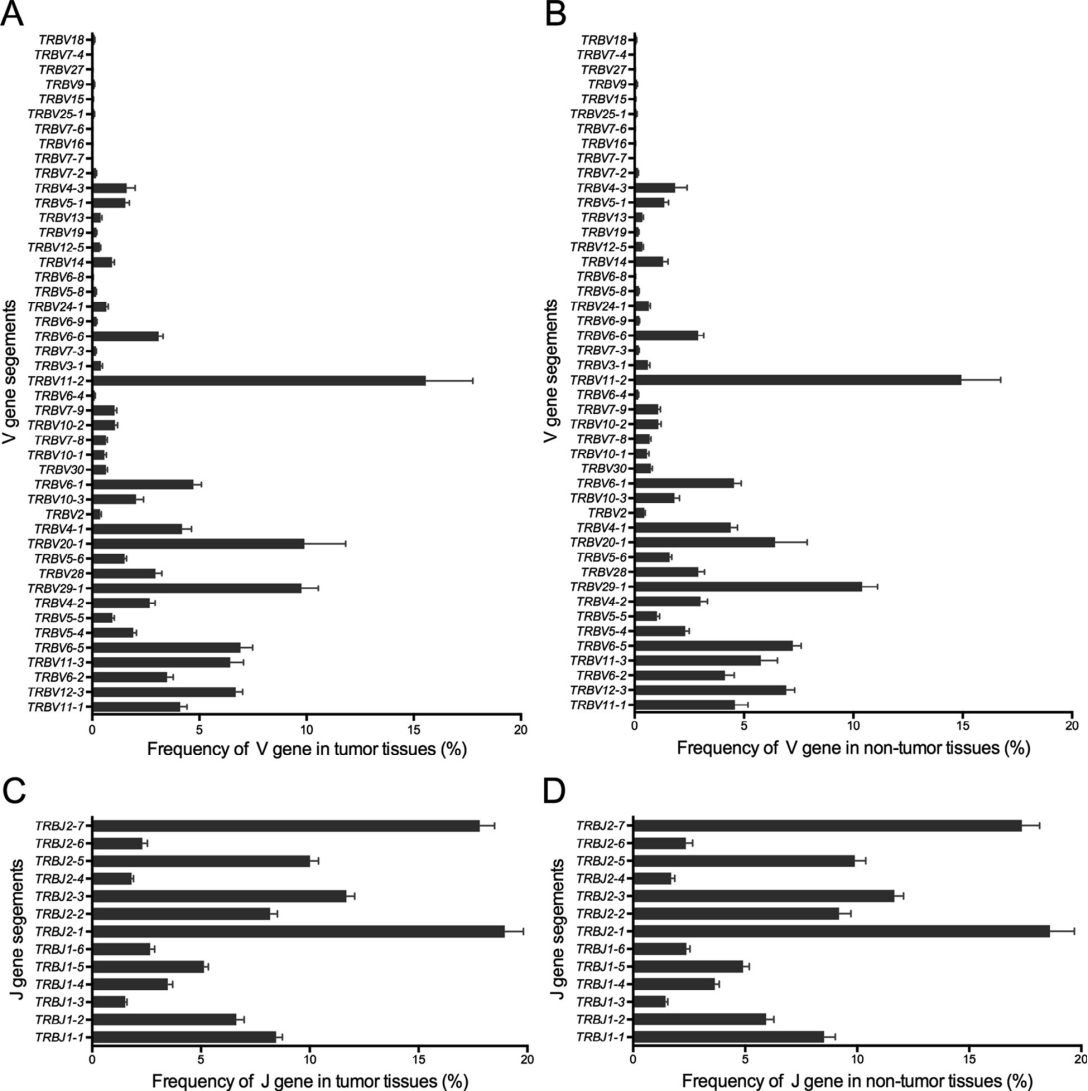
**Statistical analysis of the V and J gene usage frequencies in tumor tissues and non-tumor tissues** **A.** Frequency of V genes in tumor tissues. **B.** Frequency of V genes in non-tumor tissues. **C.** Frequency of J genes in tumor tissues. **D.** Frequency of J genes in non-tumor tissues.

**Figure 5:**
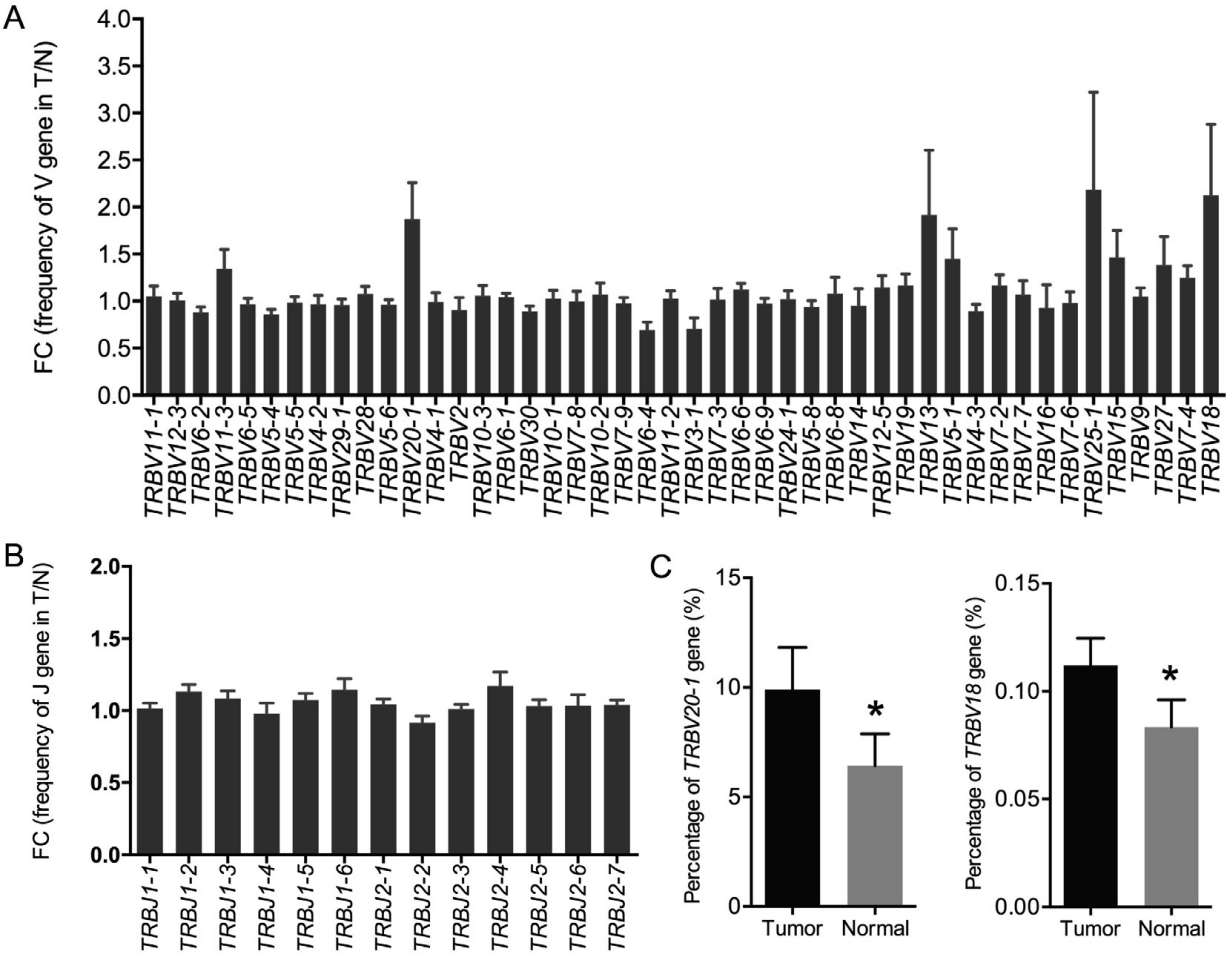
**Usage frequency of V and J gene segments between tumor tissues and normal tissues** **A.** V gene usage frequency in tumor tissues and normal tissues. **B.** J gene usage frequency in tumor tissues and normal tissues. **C.** V genes with significantly higher usage in tumor tissues than in normal tissues. FC was calculated as the mean frequency in tumor tissues divided by that in normal tissues. Frequencies with FC >1.5 or <1/1.5 were considered significant. FC, fold change. *, *P* < 0.05, *t*-test.

**Supplementary Figure S6:**
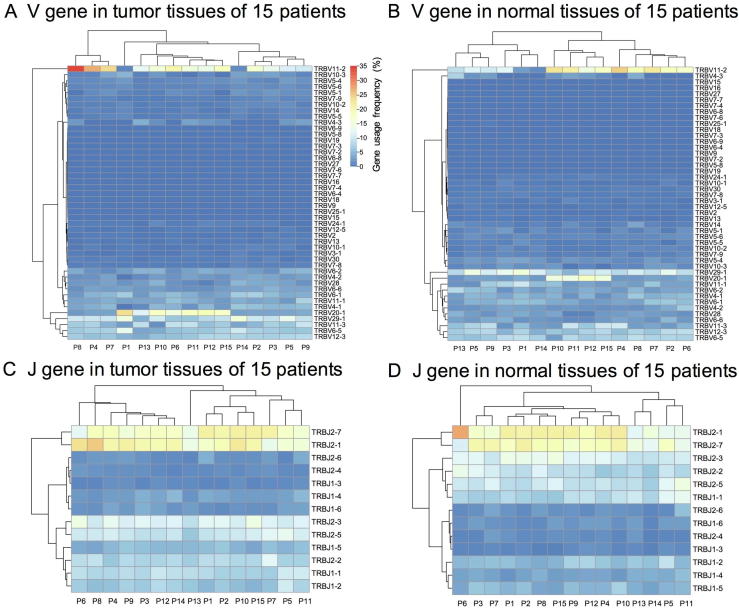
**Usage of V and J gene segments in tumor tissues and normal tissues A.** V gene usage frequency in the 15 tumor tissues. **B.** V gene usage frequency in the 15 normal tissues. **C.** J gene usage frequency in the 15 tumor tissues. **D.** J gene usage frequency in the 15 normal tissues. The color bars indicate the gene usage frequency of each sample.

Previous studies on TIL clones have suggested that the use of certain TRBV regions is predominant in tumor tissues, indicating tumor-associated antigen (TAA)-specific T cell responses [19]. In our study, based on the sequencing data, we identified 46 distinct V gene segments and 13 distinct J gene segments. We obtained 23 V gene and 2 J gene families after merging (Table S3). We evaluated the TRBV and TRBJ gene usage in the TCR repertoires of the 15 cancer tissues and normal lung tissues. Heatmap analyses of the V and J gene segment usage in these tissues showed similar usage of TRBV and TRBJ gene segments in tumor and normal lung tissues (Figure S6). Through statistical analysis and the frequencies of V and J gene usage (Figure 4), we identified the biased use of some V and J gene segments in both cancer tissues and normal lung tissues. These include TRBV11-2 (15.25%), TRBV29-1 (10.08%), TRBV20-1 (8.16%), TRBV6-5 (7.07%), and TRBV12-3 (6.81%) for V gene segments and TRBJ2-1 (18.78%), TRBJ2-7 (17.58%), TRBJ2-3 (11.67%), and TRBJ2-5 (9.95%) for J gene segments. All these genes appeared with a higher frequency than the other V and J genes in the 30 samples. We also compared the TRBV and TRBJ gene usage frequency between cancer tissues and normal lung tissues and found that TRBV20-1 and TRBV18 were used more frequently in cancer tissues than in normal lung tissues (Figure 5A, C). However, no significant difference was observed regarding TRBJ gene usage frequency between the two tissue types (Figure 5B).

## Discussion

Here, we have presented a comprehensive comparative overview of the TCRβ repertoires in lung tumor tissues and their matched normal tissues from 15 patients. The cumulative frequency of the TOP100 HECs (frequency >0.1% among all T cell clones) was higher in the normal lung tissues, indicating that T cell clones were highly expanded in normal lung tissues. The 20 clones with the highest frequencies in matched samples from each of the 15 patients also showed a higher frequency distribution in normal lung tissues than in tumor tissues. These results agree with the distribution of HECs in hepatocellular carcinoma [20], suggesting more heterogeneity and a higher T cell clonal diversity in tumor tissues than non-tumor tissues. Using blood or liver tissues, Han and colleagues [21] found it possible to distinguish liver cancer or hepatitis B patients from healthy controls by comparing HECs in blood samples, which may be used as a non-invasive detection approach for liver cancer. The authors also observed that tissues are better samples than blood for comparing diversity. In our study, we also observed significant differences of TCR repertoire diversity between cancer tissues and normal tissues. However, TCRβ repertoires in blood remain to be investigated in lung cancer to consolidate the idea that HEC ratios in blood may be a potential strategy for non-invasive cancer detection.

The host immune system involved in tumor is critical for cancer research. Immune activation against tumors is expected to prolong the survival of cancer patients. A diverse TCR repertoire is well acknowledged to be a prerequisite for an effective adaptive immune response. Many studies have investigated TCR clonal diversity among different tissues [14], [15], [22]. In colorectal tumors, studies found lower TCR clonal diversity in cancer tissues than in adjacent mucosal tissues [23]. Conversely, a significantly higher TCR diversity was found in tumor tissues than in non-tumor tissues from liver [20]. Similarly, in our study, the TCR clonal diversity in lung cancer tumor tissues was significantly higher than that in normal lung tissues, indicating that compared with tumor tissues, normal lung tissues tend toward oligoclonality. These results are consistent with our results in this paper that a higher distribution of HECs in normal lung tissues, suggesting that more antigen-associated T cell clones are generated and accumulated in normal lung tissues. The reason for the discrepancies in distinct T cell clonal diversity between different tumor tissues and peripheral tissues or blood samples might be related to the innate differences of the compared samples, specifically, the variations in the immune microenvironment in different cancer types.

In our study, a higher overlap rates were found in normal lung tissues, although the overlap rates in tumor tissues or normal lung tissues were lower than 25%. These results demonstrate a low degree of TCR repertoire similarity between these two types of tissues. The low overlap rates in tumors might indicate the intratumor heterogeneity (ITH) of lung cancer. Aging is associated with a prominent reduction in adaptive immune responses in humans. Naylor et al. investigated the effect of age on T cell generation and TCR diversity and found that the diversity of naive T cells repertoire was well maintained up to the age of 65 years but dramatically reduced afterward [24]. A recent study of TCRβ repertoires in 39 healthy donors indicated that TCRβ diversity decreased throughout life [25]. Our study of the association between TCR clonal diversity and clinicopathological characteristics of lung cancer patients also suggested that tumor tissues from younger patients (age ≤60 years old) had a higher TCR diversity than those from older patients. In normal lung tissues, younger patients tend to have higher inverse Simpson’s DIs than older patients, which was consistent with the previous study [17]. The difference was not statistically significant, more samples would be needed for further evaluation. Since there exists a reduced diversity in both tissues with ageing, we speculate that the main mechanism underlying the difference in the T cell clonal diversity between younger and older patients may be related to age-related immunosenescence. In addition, we observed that higher inverse Simpson’s DIs in tumors were associated with worse cancer outcomes. Another similar study in gastric cancer [26] demonstrated that the DI of the mucosal TCR repertoire can be a predictor of survival in gastric cancer patients, whereby a low DI was negatively correlated with patient prognosis. Therefore, the level of the diversity in T cell clones may be a promising biomarker for cancer prognosis, despite discrepancies among some studies.

Through somatic recombination of the VDJ gene segments, the α and β chain loci encode CDR3 domains that directly interact with target epitopes, producing the diversified TCR repertoires [4]. In our study, TRBV20-1 and TRBV18 were identified as differentially enriched gene segments between cancer tissues and normal lung tissues. Although the V and J gene usage patterns in lung cancer are similar to those in liver cancer [20], the differentially enriched gene segments between tumor tissues and normal lung tissues are completely different. Whether these identified genes are related to the differences in TCR repertoires between tumor tissues and matched normal lung tissues requires further study.

## Conclusion

Our study comprehensively compared the characteristics of TCR repertoires between cancer tissues and normal lung tissues in lung cancer patients. The general distribution of T cell clones was similar between cancer tissues and normal lung tissues; however, HEC rates were higher in normal lung tissues. In addition, a significantly higher TCR diversity was observed in cancer tissues than in normal lung tissues. Furthermore, TCR diversity might decrease with age, as younger patients had a higher TCR diversity than older patients. Nevertheless, further investigations using larger sample size and more detailed clinical parameters are needed for the potential use of TCR repertoires as surrogate markers for immune responses in cancer.

## Materials and methods

### Patient sample collection

Tumor tissues and the matched distant non-tumor lung tissues (>3 cm away from the edge of the tumors) with the size of 1–2 cm^3^ were obtained from 15 newly diagnosed lung cancer patients. These patients were admitted at the Department of Thoracic Surgery of Cancer Hospital of the Chinese Academy of Medical Sciences between August 2014 and November 2014 and did not receive any chemotherapy before. Exclusion criteria included previous treatment with radiotherapy or chemotherapy or suffering from other cancers at the same time. The clinical characteristics of the 15 lung cancer patients are shown in Table 1. The tissues were processed with RNAlater solution (Invitrogen, catalog No: AM7021, Carlsbad, CA) and stored at −80 °C until use. The study was reviewed and approved by the Ethics Committee of the Cancer Hospital of the Chinese Academy of Medical Sciences. Written informed consent was obtained from each patient for the current study.

**Table 1:** **Basic clinical characteristics of the 15 lung cancer patients**

**Patient ID**	**Age (year)**	**Gender**	**Pathological type**	**Differentiation**	**TNM stage**
P1	62	Male	ADC	L	T3N2M0 (III)
P2	56	Female	ADC	L	T2bN0M0 (I)
P3	57	Male	ADC	L	T2aN0M0 (I)
P4	43	Female	ADC	H	T1N0M0 (I)
P5	73	Male	SCC	L	T2aN2M0 (III)
P6	65	Male	SCLC	–	T2aN0M0 (I)
P7	76	Male	SCC	M	T1aN1M0 (II)
P8	73	Male	ADC	L	T2aN2M0 (III)
P9	64	Male	ADC	L	T2bN0M0 (II)
P10	56	Female	ADC	M	T2aN0M0 (I)
P11	64	Male	ADC	M	T1N0M0 (I)
P12	31	Male	ADC	L	T3N2M0 (III)
P13	61	Female	ADC	M	T2N0M0 (I)
P14	73	Female	ADC	H	T1aN0M0 (I)
P15	41	Male	ADC	L	T2N2M0 (III)

*Note*: ADC, adenocarcinoma; SCC, squamous cell carcinoma; SCLC, small cell lung cancer; L, poorly differentiated; M, moderately differentiated; H, well differentiated.

### TCRβ sequencing and data analysis

For each sample, RNA was extracted and evaluated by NanoDrop ND-1000 as well as Agilent 2100. Then, three rounds of nested PCR were performed using primers (Table S4) according to protocols modified from ARM-PCR [27], [28] for the construction of TCR sequencing libraries as described previously [15], [18]. After three rounds of nested PCR, the PCR product was separated on gel and bands of 200–500 bp were excised for purification using the QIAquick gel extraction kit (Qiagen, catalog No: 28706, Hilden, Germany). The purified DNA product was then sequenced.

We used Trimmomatic [29] to acquire clean data and FLASH [30] to get complete TCRβ CDR3 sequences. Then, rearranged mRNA sequences were assigned to their germline VDJ counterparts using MiTCR [31], [32]. VDJtools was used for statistical analysis of T cell clones (calculated using inverse Simpson’s DI) [33].

### Statistical analysis

To compare paired tumor tissues and normal lung tissues, Wilcoxon signed rank test (two-tailed) was used. We used Mann-Whitney U test (two-tailed) to compare TCR diversity among samples and its association with different clinicopathological characteristics, whereas paired *t*-tests were used to identify differentially enriched gene segments. A Kaplan-Meier plot with log-rank test was employed to compare survival among groups. Statistical analyses were conducted through GraphPad Prism 6, Microsoft Excel and R biostatistical software. *P* < 0.05 was considered statistically significant.

## Data availability

The raw data for TCR repertoire sequencing have been deposited in the NCBI Sequence Read Archive database (SRA) as SRA: SRP126857.

## Authors’ contributions

XW constructed the TCR library and performed the data analysis. BZ participated in acquisition of data. YY, JZ, and YM participated in the collection of clinical samples and the acquisition of data. SC coordinated the design of the study. LF analyzed the raw data. TX designed the study. XW drafted the manuscript with the help of TX. All authors read and approved the final manuscript.

## Competing interests

The authors have declared no competing interests.
