# Supplementary material for: Characterization of Distinct T Cell Receptor Repertoires in Tumor and Distant Non-tumor Tissues from Lung Cancer Patients
Source: Genomics Proteomics Bioinformatics. 2019 Aug 31;17(3):287–96. doi: 10.1016/j.gpb.2018.10.005 (PMC6818398; doi:10.1016/j.gpb.2018.10.005)
Supplement: Supplementary Table S1 [file mmc1.docx]

**Table S1 Statistical characteristics** **of the TCRβ CDR3 repertoires data in 15 lung cancer patients**

| **ID** | **Sample** | **No. of productive reads** | **No. of unique clones** | **Highest frequency clones (%)** | **No. of unique V** | **No. of unique D** | **No. of unique J** | **TOP100 clone (%)** | **No. of unique CDR3 (nt)** | **No. of unique CDR3 (aa)** | **Inverse Simpson’s DI** |
| --- | --- | --- | --- | --- | --- | --- | --- | --- | --- | --- | --- |
| P1 | T | 3,015,213 | 370,128 | 7.43 | 46 | 2 | 13 | 34.60 | 366,918 | 244,078 | 129.44 |
|  | N | 5,758,840 | 413,782 | 13.60 | 46 | 2 | 13 | 48.50 | 408,290 | 259,919 | 40.60 |
| P2 | T | 3,706,392 | 468,181 | 3.18 | 46 | 2 | 13 | 24.42 | 463,659 | 305,915 | 569.15 |
|  | N | 5,240,541 | 545,296 | 4.15 | 46 | 2 | 13 | 31.64 | 540,224 | 350,455 | 282.49 |
| P3 | T | 4,178,684 | 400,669 | 2.34 | 46 | 2 | 13 | 35.83 | 395,843 | 270,780 | 312.60 |
|  | N | 3,818,744 | 274,202 | 11.51 | 46 | 2 | 13 | 50.29 | 271,357 | 179,748 | 44.77 |
| P4 | T | 3,826,411 | 528,980 | 0.96 | 46 | 2 | 13 | 22.63 | 523,364 | 347,113 | 1118.66 |
|  | N | 3,428,061 | 316,427 | 3.25 | 46 | 2 | 13 | 33.66 | 312,780 | 211,292 | 316.68 |
| P5 | T | 5,278,951 | 630,911 | 3.47 | 46 | 2 | 13 | 32.74 | 624,022 | 398,072 | 309.32 |
|  | N | 2,639,987 | 373,768 | 10.23 | 46 | 2 | 13 | 40.20 | 367,916 | 241,269 | 73.84 |
| P6 | T | 3,618,382 | 570,527 | 3.45 | 46 | 2 | 13 | 27.35 | 560,665 | 399,318 | 316.16 |
|  | N | 4,304,604 | 503,417 | 6.28 | 46 | 2 | 13 | 36.15 | 498,515 | 328,637 | 124.08 |
| P7 | T | 5,288,808 | 522,309 | 2.04 | 46 | 2 | 13 | 32.05 | 516,911 | 348,790 | 472.12 |
|  | N | 4,479,280 | 368,437 | 5.43 | 46 | 2 | 13 | 42.54 | 364,233 | 244,736 | 117.18 |
| P8 | T | 5,733,528 | 331,272 | 28.79 | 46 | 2 | 13 | 55.15 | 328,127 | 218,968 | 11.75 |
|  | N | 4,688,981 | 323,035 | 6.01 | 46 | 2 | 13 | 44.10 | 320,636 | 209,710 | 106.22 |
| P9 | T | 5,385,617 | 580,629 | 2.28 | 46 | 2 | 13 | 29.53 | 573,555 | 392,711 | 402.56 |
|  | N | 5,797,795 | 615,647 | 3.82 | 46 | 2 | 13 | 30.79 | 608,613 | 423,713 | 285.06 |
| P10 | T | 3,638,226 | 533,319 | 1.62 | 46 | 2 | 13 | 23.72 | 529,851 | 349,336 | 778.27 |
|  | N | 3,665,899 | 414,089 | 3.08 | 46 | 2 | 13 | 40.45 | 410,157 | 273,607 | 204.81 |
| P11 | T | 3,436,238 | 704,296 | 8.41 | 46 | 2 | 13 | 26.93 | 690,842 | 501,945 | 117.31 |
|  | N | 3,975,429 | 434,191 | 14.92 | 46 | 2 | 13 | 45.86 | 426,482 | 276,866 | 36.96 |
| P12 | T | 3,241,650 | 523,322 | 2.08 | 46 | 2 | 13 | 24.47 | 518,170 | 365,291 | 694.48 |
|  | N | 3,598,545 | 515,841 | 3.59 | 46 | 2 | 13 | 27.97 | 511,369 | 357,077 | 327.83 |
| P13 | T | 3,759,321 | 526,833 | 6.82 | 46 | 2 | 13 | 36.75 | 517,857 | 348,942 | 105.81 |
|  | N | 2,837,833 | 330,543 | 3.19 | 46 | 2 | 13 | 35.02 | 327,229 | 217,876 | 218.31 |
| P14 | T | 3,198,192 | 439,725 | 1.41 | 46 | 2 | 13 | 24.31 | 435,427 | 299,351 | 762.10 |
|  | N | 3,231,565 | 310,974 | 4.07 | 46 | 2 | 13 | 41.35 | 307,068 | 206,055 | 148.85 |
| P15 | T | 5,251,813 | 727,815 | 3.71 | 46 | 2 | 13 | 26.21 | 720,099 | 489,815 | 370.79 |
|  | N | 5,052,378 | 526,377 | 5.47 | 46 | 2 | 13 | 33.75 | 519,774 | 350,618 | 165.34 |

*Note*: T, tumor tissue; N, non-tumor normal tissue; CDR3, complementarity determining region 3; DI, diversity index. Highest frequency clones (%), the percentage for the clone with highest frequency in each sample.
