# Supplementary material for: Characterization of Distinct T Cell Receptor Repertoires in Tumor and Distant Non-tumor Tissues from Lung Cancer Patients
Source: Genomics Proteomics Bioinformatics. 2019 Aug 31;17(3):287–96. doi: 10.1016/j.gpb.2018.10.005 (PMC6818398; doi:10.1016/j.gpb.2018.10.005)
Supplement: Supplementary Table S2 [file mmc2.docx]

**Table S2** **Summary of TCRβ CDR3 clones with different frequencies in 15 lung cancer patients**

| **Patient ID** | **Sample** | **TCRβ clone frequency (%)** | | | | | |
| --- | --- | --- | --- | --- | --- | --- | --- |
|  |  | **< 0.0001%** | **0.0001%–0.001%** | **0.001%–0.01%** | **0.01%–0.1%** | **0.1%–1%** | **> 1%** |
| P1 | T | 87.75828902 | 10.19025851 | 1.781545844 | 0.247752129 | 0.02026326 | 0.001891238 |
|  | N | 90.41306775 | 8.133993262 | 1.244858404 | 0.188263385 | 0.018125486 | 0.001691712 |
| P2 | T | 86.19956812 | 12.06328322 | 1.500274466 | 0.218932421 | 0.017728186 | 0.000213593 |
|  | N | 90.70119715 | 7.85408292 | 1.240977377 | 0.186870984 | 0.015771251 | 0.00110032 |
| P3 | T | 87.8910023 | 10.21316848 | 1.659224946 | 0.21239477 | 0.022212849 | 0.001996661 |
|  | N | 84.96765159 | 12.77561798 | 1.931058125 | 0.292120408 | 0.031363739 | 0.002188168 |
| P4 | T | 86.70460131 | 11.60875647 | 1.467919392 | 0.201708949 | 0.017013876 | 0 |
|  | N | 84.12177216 | 13.47514593 | 2.033012354 | 0.33183009 | 0.036659324 | 0.001580143 |
| P5 | T | 91.71515475 | 7.003047973 | 1.12614933 | 0.142492364 | 0.012204574 | 0.000951006 |
|  | N | 85.24833587 | 12.84807688 | 1.681791914 | 0.202799598 | 0.017658012 | 0.001337728 |
| P6 | T | 87.95149046 | 10.51746894 | 1.36119763 | 0.159151101 | 0.009990763 | 0.000701106 |
|  | N | 90.3712032 | 8.059918517 | 1.366858886 | 0.190100851 | 0.010528051 | 0.001390497 |
| P7 | T | 90.01606329 | 8.356164646 | 1.447993429 | 0.161781627 | 0.017231179 | 0.00076583 |
|  | N | 87.93335088 | 10.19767287 | 1.617915682 | 0.227447298 | 0.021170512 | 0.002442751 |
| P8 | T | 89.49473544 | 8.916841749 | 1.369267551 | 0.192590983 | 0.025356806 | 0.001207467 |
|  | N | 87.46080146 | 10.50288668 | 1.714365316 | 0.29253796 | 0.026622502 | 0.002786076 |
| P9 | T | 90.55972058 | 7.801194911 | 1.432756545 | 0.190999761 | 0.014294842 | 0.001033362 |
|  | N | 90.76451278 | 7.658771991 | 1.411685593 | 0.154471637 | 0.009908275 | 0.000649723 |
| P10 | T | 87.33928474 | 11.20605116 | 1.242970905 | 0.195755261 | 0.015375413 | 0.000562515 |
|  | N | 88.00620157 | 10.1222201 | 1.667998908 | 0.181603472 | 0.019561012 | 0.00241494 |
| P11 | T | 89.6052228 | 9.190595999 | 1.08931472 | 0.107483217 | 0.006957302 | 0.000425957 |
|  | N | 88.62643399 | 9.895875318 | 1.301270639 | 0.158916237 | 0.016121937 | 0.00138188 |
| P12 | T | 88.04005947 | 10.13200286 | 1.651564429 | 0.161659552 | 0.014140434 | 0.000573261 |
|  | N | 88.53600237 | 9.468033755 | 1.804819702 | 0.176604807 | 0.013376215 | 0.001163149 |
| P13 | T | 88.75545002 | 9.989693129 | 1.082885848 | 0.160012755 | 0.010819368 | 0.001138881 |
|  | N | 81.63567221 | 16.16340385 | 1.859969807 | 0.316751527 | 0.02178234 | 0.00242026 |
| P14 | T | 87.34686452 | 10.50292797 | 1.898686679 | 0.233100233 | 0.017738359 | 0.000682245 |
|  | N | 86.51302038 | 10.95075473 | 2.22430171 | 0.287483841 | 0.02154521 | 0.002894133 |
| P15 | T | 92.10499921 | 6.666117076 | 1.089699992 | 0.129978085 | 0.008793443 | 0.000412193 |
|  | N | 91.21561162 | 7.108593271 | 1.476508282 | 0.184468546 | 0.013868387 | 0.00094989 |
