# Supplementary material for: Characterization of Distinct T Cell Receptor Repertoires in Tumor and Distant Non-tumor Tissues from Lung Cancer Patients
Source: Genomics Proteomics Bioinformatics. 2019 Aug 31;17(3):287–96. doi: 10.1016/j.gpb.2018.10.005 (PMC6818398; doi:10.1016/j.gpb.2018.10.005)
Supplement: Supplementary Table S3 [file mmc3.docx]

**Table S3 Merged TRBV and TRBJ gene segments**

| **Vβ or Jβ** | **Segment** | **Family** |
| --- | --- | --- |
| **Vβ** | TRBV2 | TRBV2 |
|  | TRBV3-1 | TRBV3 |
|  | TRBV4-1 | TRBV4 |
|  | TRBV4-2 |  |
|  | TRBV4-3 |  |
|  | TRBV5-1 | TRBV5 |
|  | TRBV5-4 |  |
|  | TRBV5-5 |  |
|  | TRBV5-6 |  |
|  | TRBV5-8 |  |
|  | TRBV6-1 | TRBV6 |
|  | TRBV6-2 |  |
|  | TRBV6-4 |  |
|  | TRBV6-5 |  |
|  | TRBV6-6 |  |
|  | TRBV6-8 |  |
|  | TRBV6-9 |  |
|  | TRBV7-2 | TRBV7 |
|  | TRBV7-3 |  |
|  | TRBV7-4 |  |
|  | TRBV7-6 |  |
|  | TRBV7-7 |  |
|  | TRBV7-8 |  |
|  | TRBV7-9 |  |
|  | TRBV8-2 | TRBV8 |
|  | TRBV9 | TRBV9 |
|  | TRBV10-1 | TRBV10 |
|  | TRBV10-2 |  |
|  | TRBV10-3 |  |
|  | TRBV11-1 | TRBV11 |
|  | TRBV11-2 |  |
|  | TRBV11-3 |  |
|  | TRBV12-3 | TRBV12 |
|  | TRBV12-5 |  |
|  | TRBV13 | TRBV13 |
|  | TRBV14 | TRBV14 |
|  | TRBV15 | TRBV15 |
|  | TRBV16 | TRBV16 |
|  | TRBV18 | TRBV18 |
|  | TRBV19 | TRBV19 |
|  | TRBV24-1 | TRBV24 |
|  | TRBV25-1 | TRBV25 |
|  | TRBV27 | TRBV27 |
|  | TRBV28 | TRBV28 |
|  | TRBV29-1 | TRBV29 |
|  | TRBV30 | TRBV30 |
|  | 46 Vβ segments in total | 23 Vβ families in total |
| **Jβ** | TRBJ1-1 | TRBJ1 |
|  | TRBJ1-2 |  |
|  | TRBJ1-3 |  |
|  | TRBJ1-4 |  |
|  | TRBJ1-5  TRBJ1-6 |  |
|  | TRBJ2-1 | TRBJ2 |
|  | TRBJ2-2 |  |
|  | TRBJ2-3 |  |
|  | TRBJ2-4 |  |
|  | TRBJ2-5  TRBJ2-6 |  |
|  | TRBJ2-7 |  |
|  | 13 Jβ segments in total | 2 Jβ families in total |
